# Supplementary material for: Comparison of Secular Trends in Peptic Ulcer Diseases Mortality in China, Brazil and India during 1990–2019: An Age-Period-Cohort Analysis
Source: Healthcare (Basel). 2023 Apr 11;11(8):1085. doi: 10.3390/healthcare11081085 (PMC10137755; doi:10.3390/healthcare11081085)
Supplement: Supplementary file 1 [file healthcare-11-01085-s001.zip › healthcare-2194033-supplementary.pdf]

Table S1. Wald Chi Square tests for estimable functions in the APC model.

Null Hypothesis	China		Brazil		India	
	Chi-Square	P-Value	Chi-Square	P-Value	Chi-Square	P-Value
Net Drift = 0	3616.09	<0.001	6559.95	<0.001	4359.21	<0.001
All Period RR = 1	3685.36	<0.001	6747.69	<0.001	4454.74	<0.001
All Cohort RR = 1	9662.80	<0.001	12769.52	<0.001	5968.99	<0.001
All Local Drifts = Net Drift	481.11	<0.001	154.73	<0.001	55.51	<0.001

Table S2. The local drifts for China, Brazil and India by gender (%)

Age	China-M	China-F	Brazil-M	Brazil-F	India-M	India-F
17.5	-5.87 (-7.56 to -4.15)	-6.51 (-9.73 to -3.18)	-2.63 (-3.51 to -1.73)	-3.43 (-4.87 to -1.97)	-6.21 (-7.18 to -5.24)	-6.56 (-7.64 to -5.47)
22.5	-5.84 (-6.78 to -4.90)	-7.24 (-9.08 to -5.36)	-3.60 (-4.16 to -3.03)	-3.84 (-4.78 to -2.88)	-5.59 (-6.22 to -4.96)	-5.83 (-6.50 to -5.16)
27.5	-5.66 (-6.25 to -5.06)	-7.69 (-8.90 to -6.45)	-4.34 (-4.75 to -3.92)	-4.44 (-5.15 to -3.71)	-5.38 (-5.82 to -4.93)	-5.22 (-5.76 to -4.68)
32.5	-5.22 (-5.66 to -4.78)	-7.29 (-8.24 to -6.33)	-4.68 (-4.99 to -4.36)	-4.67 (-5.22 to -4.11)	-5.30 (-5.66 to -4.93)	-4.54 (-5.02 to -4.06)
37.5	-5.02 (-5.36 to -4.67)	-6.85 (-7.62 to -6.07)	-4.71 (-4.96 to -4.46)	-4.60 (-5.04 to -4.16)	-5.21 (-5.52 to -4.91)	-4.06 (-4.50 to -3.61)
42.5	-5.06 (-5.31 to -4.80)	-6.59 (-7.18 to -5.98)	-4.48 (-4.69 to -4.27)	-4.33 (-4.69 to -3.96)	-4.99 (-5.25 to -4.73)	-3.69 (-4.10 to -3.28)
47.5	-5.16 (-5.37 to -4.96)	-6.41 (-6.90 to -5.92)	-4.17 (-4.35 to -4.00)	-3.85 (-4.15 to -3.55)	-5.03 (-5.26 to -4.80)	-3.73 (-4.10 to -3.36)
52.5	-5.25 (-5.43 to -5.08)	-6.32 (-6.74 to -5.90)	-3.88 (-4.04 to -3.73)	-3.51 (-3.76 to -3.26)	-5.07 (-5.29 to -4.86)	-3.69 (-4.02 to -3.36)
57.5	-5.30 (-5.46 to -5.14)	-6.20 (-6.57 to -5.83)	-3.78 (-3.92 to -3.64)	-3.33 (-3.55 to -3.12)	-5.22 (-5.41 to -5.02)	-3.98 (-4.27 to -3.69)
62.5	-4.98 (-5.12 to -4.84)	-5.68 (-5.99 to -5.38)	-3.67 (-3.81 to -3.54)	-3.38 (-3.57 to -3.19)	-5.24 (-5.43 to -5.06)	-4.03 (-4.30 to -3.77)
67.5	-4.54 (-4.67 to -4.41)	-5.16 (-5.42 to -4.90)	-3.57 (-3.70 to -3.45)	-3.44 (-3.62 to -3.26)	-5.05 (-5.23 to -4.88)	-4.02 (-4.26 to -3.77)
72.5	-4.05 (-4.18 to -3.92)	-4.57 (-4.81 to -4.33)	-3.41 (-3.54 to -3.28)	-3.41 (-3.58 to -3.24)	-4.70 (-4.89 to -4.51)	-3.92 (-4.17 to -3.67)
77.5	-3.53 (-3.68 to -3.39)	-3.90 (-4.13 to -3.66)	-3.25 (-3.40 to -3.10)	-3.34 (-3.51 to -3.17)	-4.44 (-4.66 to -4.21)	-3.88 (-4.16 to -3.59)
82.5	-3.02 (-3.22 to -2.82)	-3.25 (-3.55 to -2.94)	-3.13 (-3.33 to -2.93)	-3.38 (-3.60 to -3.16)	-4.04 (-4.38 to -3.69)	-3.65 (-4.05 to -3.25)
